# Effects of a multifaceted intervention on cardiovascular risk factors in high-risk hypertensive patients: the ESCAPE trial, a pragmatic cluster randomized trial in general practice

**DOI:** 10.1186/1745-6215-14-318

**Published:** 2013-10-01

**Authors:** Denis Pouchain, Michel Lièvre, Dominique Huas, Jean-Pierre Lebeau, Vincent Renard, Eric Bruckert, Xavier Girerd, Florent Boutitie

**Affiliations:** 1 Collège National des Généralistes Enseignants, 6 bis, rue des Deux Communes, Vincennes, 94300, France; 2 Département de Médecine Générale, Faculté de Médecine, Université François Rabelais, 10, boulevard Tonnellé, BP 3223, , Tours Cedex 1, 37032, France; 3 Service de Pharmacologie Clinique, Université Claude Bernard, CNRS UMR 5558, rue Guillaume Paradin, BP 8071, Lyon Cedex 08, 69376,, France; 4 Département de Médecine Générale, Faculté de Médecine, Université Paris-Est Créteil Val de Marne (UPEC), 8 avenue du Général Sarrail, Créteil Cedex, 94010,, France; 5 Service d’Endocrinologie-Métabolisme, Hôpital de la Pitié-Salpêtrière, 45-83 boulevard de l’Hôpital, Paris Cedex 13, 75651,, France; 6 Hospices Civils de Lyon, Centre Hospitalier Lyon-Sud. Service de Biostatistiques, CNRS UMR 5558, rue Guillaume Paradin, BP 8071, Lyon Cedex 8, 69376,, France

**Keywords:** Hypertension, Primary prevention, Cluster randomized trial, General practice

## Abstract

**Background:**

Several observational studies on hypertensive patients have shown a gap between therapeutic targets recommended in guidelines and those achieved in daily practice. The ESCAPE trial aimed to determine whether a multifaceted intervention focused on general practitioners (GPs), could increase significantly the proportion of hypertensive patients at high risk in primary prevention who achieved all their recommended therapeutic targets.

**Methods:**

A pragmatic, cluster randomized trial involving 257 GPs randomized by region. The GPs in the intervention group had a one-day training session and were given an electronic blood pressure measurement device and a short recommendation leaflet. Along with usual follow-up, they focused one consultation on hypertension and other cardiovascular risk factors every six months for two years. They also received feedback at baseline and at one year on their patients’ clinical and biological parameters. Main outcome measures were change in the proportion of patients achieving all their therapeutic targets and each individual therapeutic target at two years, and quality of life.

**Results:**

1,832 high-risk hypertensive patients were included. After two years, the proportion of patients achieving all their therapeutic targets increased significantly in both groups, but significantly more in the intervention group: OR (odds-ratio) 1.89, (95% confidence interval (CI) 1.09 to 3.27, *P* = 0.02). Significantly more patients achieved their blood pressure targets in the intervention group than in the usual care group: OR 2.03 (95% CI 1.44 to 2.88, *P* < 0.0001). Systolic and diastolic blood pressures decreased significantly more in the intervention group than in the usual care group, by 4.8 mmHg and 1.9 mmHg, respectively (*P* < 0.0001 for both). There were no significant difference changes in physical and mental quality of life between groups.

**Conclusion:**

An easy-to-perform, multifaceted intervention targeting only GPs increased significantly the proportion of high-risk hypertensive patients in primary prevention achieving their recommended therapeutic targets.

**Trial registration:**

This trial was registered with ClinicalTrials.gov, number NCT00348855

## Background

French [1,2] and European [3] guidelines for the primary prevention of cardiovascular complications in patients with hypertension are mainly based on blood pressure targets that should be achieved along with other risk-reducing strategies. As these patients are at an increased cardiovascular risk, the recommendations also set targets for low-density lipoprotein cholesterol (LDL), smoking cessation, and, in patients with type-2 diabetes, HbA1c and low-dose aspirin treatment [2].

In France, more than ten million patients are treated for hypertension [4]. Slightly more than three-quarters of these are for primary prevention [5] and 92% are followed exclusively by general practitioners (GPs) [4]. Recent studies in the general population [6,7] have reported that between 50% and 76% of treated hypertensive patients had uncontrolled hypertension, that is, ≥ 140/90 mmHg. For those followed by GPs, the last published rate was 58%, showing a gap between recommendations and practice [8]. Furthermore, the more risk factors that hypertensive patients receiving primary prevention treatment have, the worse these risk factors are controlled [5,9,10].

A common explanation for the gap between guidelines and practice is patients’ lack of adherence, but another key reason is therapeutic inertia, that is, the failure of health care providers to start or increase treatment when the therapeutic targets are not met [11,12]. Targeting an intervention to the healthcare providers is one means of ensuring that patients receive optimal therapeutic benefit by reducing therapeutic inertia. Randomized trials undertaken in this area have evaluated various types of interventions (mono- or multifaceted) with patients, their families, physicians, physician assistants, nurses [13], pharmacists and health care organizations, either separately or in different combinations. A systematic review of these trials reported a non-significant reduction of 0.4 mmHg (95% confidence interval (CI) = -1.1 to 0.2) in systolic blood pressure (SBP) and 0.4 mmHg (95% CI = -1.1 to 0.3) in diastolic blood pressure (DBP) in patients followed by healthcare providers who were randomized to the intervention groups [14].

The few randomized clinical trials that measured the impact of interventions targeting GPs, were heterogeneous in their modalities and in most cases; other health professionals (for example, nurses, assistants) also received the intervention. These interventions did not significantly increase the proportion of patients achieving recommended therapeutic targets or a clinically relevant reduction in blood pressure [12-18].

The ESCAPE trial aimed to determine whether a multifaceted intervention focused exclusively on GPs could increase significantly the proportion of hypertensive patients at high risk in primary prevention who achieved their recommended therapeutic targets.

## Methods

ESCAPE was a two-year pragmatic, randomized cluster controlled trial. In fact, a traditional randomized trial in which the patients would have been randomized was impossible because the intervention was aimed at doctors, not patients. The Institutional Review Board of Versailles approved the study in May 2006. All patients gave written informed consent for their data to be used for this trial.

### Participants

The physicians were all GPs and were members of the French National College of Teachers in General Practice (CNGE). Firstly, all the 33 French regional colleges belonging to the CNGE were invited to participate in 'a randomized trial with hypertensive patients’. Twenty-three of these colleges agreed to participate. Eight hundred and seventy-seven GPs, members of these 23 colleges, were contacted by each regional research leader by telephone and/or Email, or during a usual meeting, and 335 agreed to participate.

### Randomization

One of our aims was to reduce the contamination bias, which corresponds to the appropriation of the intervention group methods by the control group, following contacts between investigators. To minimize contamination bias as much as possible, the regional colleges were randomized rather than the GPs themselves. We achieved therefore a sort of geographical and functional isolation of the clusters. Thus, all participating GPs from a given regional college were randomized to the same study group. Randomization was conducted after recruiting GPs [19] through the use of a computer program provided by the Claude Bernard University Department of Clinical Pharmacology in Lyon that was also in charge of the data management. Of the 23 colleges which agreed to participate in the trial, 11 were allocated to the usual care group and 12 to the intervention group.

### Patients’ inclusion criteria

To be included, patients had to be aged between 45 and 75 years, to be treated for hypertension for at least six months, not to have any known clinical signs or history of cardiovascular disease, and to have at least two of the following cardiovascular risk factors [1]:

•Age ≥ 50 years for men and ≥ 60 years for women.

•Family history of myocardial infarction or sudden early death (at ≤ 55 years for a first-degree male relative or ≤ 65 years for a first-degree female relative) or stroke at ≤ 45 years for a first-degree relative.

•Active smoker or having quit smoking < three years ago.

•Treated or untreated type-2 diabetes (fasting glycemia ≥ 7 mmol/L at two occurrences or use of an anti-diabetic drug).

•LDL ≥ 4.14 mmol/L or use of lipid-lowering drug.

•High-density lipoprotein cholesterol (HDL) ≤ 1.04 mmol/L (one risk factor was subtracted if HDL ≥ 1.55 mmol/L).

•Known left ventricular hypertrophy (diagnosed by ultrasound or electrocardiography).

•Urinary excretion of albumin ≥ 20 mg/L.

GPs were asked to include the first eligible patients they saw over a week, with a minimum of seven patients.

### Exclusion criteria

Patients were not eligible if they had type-1 diabetes, were unable to participate in a two-year trial, had a serious life-threatening disease with a poor short-term prognosis, or could not understand French.

### Intervention

In the intervention group, GPs attended a one-day training seminar about therapeutic targets and strategies to achieve them as recommended by the French guidelines [1,2]. Using a common teaching kit, four trained university GP lecturers delivered the standardized regional training seminars between September and December 2006. The GPs in this group were given a validated electronic blood pressure measurement device (Spengler TB101, Spengler SAS, Antony, France) to improve the accuracy of blood pressure measurements. They were also given a six-page leaflet that summarized targets and therapeutic strategies recommended in the guidelines which they were asked to keep on their office desk.

Every six months during the two-year trial, the GPs were asked to dedicate one routine follow-up consultation to optimize (if needed and possible) the treatment of the patients who had not achieved their individual targets. The GPs were also asked to discuss systematically the patient’s lifestyle (diet and exercise), adherence to drug treatment and to give advice on quitting smoking if the patient smoked. Advice for lifestyle was not standardized. Lastly, at the end of recruitment and after the 12-month consultation, GPs in the intervention group received feedback on their patients’ clinical and biological data.

The GPs randomized in the usual care group attended a 90-minute meeting to learn about the inclusion and exclusion criteria and how to complete the study case report forms. They were not told about the study aims, the nature of the intervention or any endpoints, so that they would continue to treat their patients in their usual way.

### Patient follow-up

At baseline, and every six months for two years along with usual follow-up, the GPs in both groups collected patients’ clinical and biological data. Prescriptions of drugs for hypertension and metabolic treatment were reported at baseline and 24 months. At inclusion, 12-months and 24-months, patients of both groups were given a sealed envelope containing five short questionnaires on quality of life (SF-8), adherence [20], diet, exercise, and smoking habits to be completed at home and sent directly to the data treatment center in a pre-paid envelope. The patients could therefore complete the questionnaires without being influenced by their doctor, hence by the group to which they belonged.

### Endpoints

The primary endpoint was the change in the proportion of patients achieving all of their therapeutic targets at two years. Three therapeutic targets were defined for patients without type-2 diabetes: BP ≤ 140/90 mmHg, LDL ≤ 3.36 mmol/l, and no smoking [1]. Five therapeutic targets were defined for patients with type-2 diabetes: BP ≤ 130/80 mmHg, LDL ≤ 2.59 mmol/l, HbA1c ≤ 7%, no smoking, and a prescription for low-dose aspirin [2].

The secondary endpoints were the change in the proportion of patients achieving each of their individual targets and the values for BP, LDL, and HbA1c. Other secondary endpoints were the variation in the Framingham-Anderson score for coronary risk [21], the occurrence of the first clinical cardiovascular event (validated by a committee blinded to randomization), change in antihypertensive drug prescriptions, and quality of life.

### Sample size calculation

The sample size was calculated following the method of Hayes and Bennett [22], accounting for the cluster design of the trial. This method uses the between-cluster coefficient of variation k, which was estimated to be 0.27 at the end of the inclusion period. For the primary endpoint in the whole included population, it was calculated that 23 clusters including 70 patients each, would be needed to give the trial a 90% power to detect success rate of 10.5% in the usual care group and 19.5% in the intervention group, assuming a conservative value 0.3 for k, and an alpha risk of 0.05. Assuming that 10% of the data would not be available at the end of the trial, it was calculated that 885 patients were necessary in each study group.

### Statistical analyses

The intervention and usual care groups were compared at baseline using the Kruskal-Wallis test for continuous variables, the Fisher exact test for binary variables, and the chi-squared test for categorical variables with more than two categories.

Three-level hierarchical generalized linear models were used to analyze the evolution of the criteria over time, with practices and patients included as random effects. The model also included a fixed group effect, a linear time effect, and a differential effect of the intervention group versus the usual care group over time (interaction), which was the effect of interest. Missing outcomes, at a given time point, were estimated with their unbiased linear predictor. Another fixed-effect model was used to estimate directly the evolution of the criteria over time in the two groups (with 95% confidence intervals). Continuous and binary variables were modeled using an identity function and a logit link function, respectively. The results are presented as odds-ratios (OR) with 95% CI, and reported at the patient level.

A *post hoc* sensitivity analysis restricted to centers that used only an automatic blood pressure measurement device was performed to evaluate the extent to which the use of conventional blood pressure measurement in the usual care group might have affected the results. We performed another *post hoc* analysis of blood pressure changes that included all the patient population and was adjusted on baseline blood pressure.

The data preparation and descriptive statistics were performed using SAS (Windows, v. 9.1, SAS Institute Inc., Cary, NC, USA.). Modeling was performed using the generalized linear latent and mixed models (gllamm) function of STATA (Windows, v. 9, StataCorp. 2005. /Stata Statistical Software: Release 9/. College Station, TX: StataCorp LP).

## Results

### Recruitment

**Figure 1:**
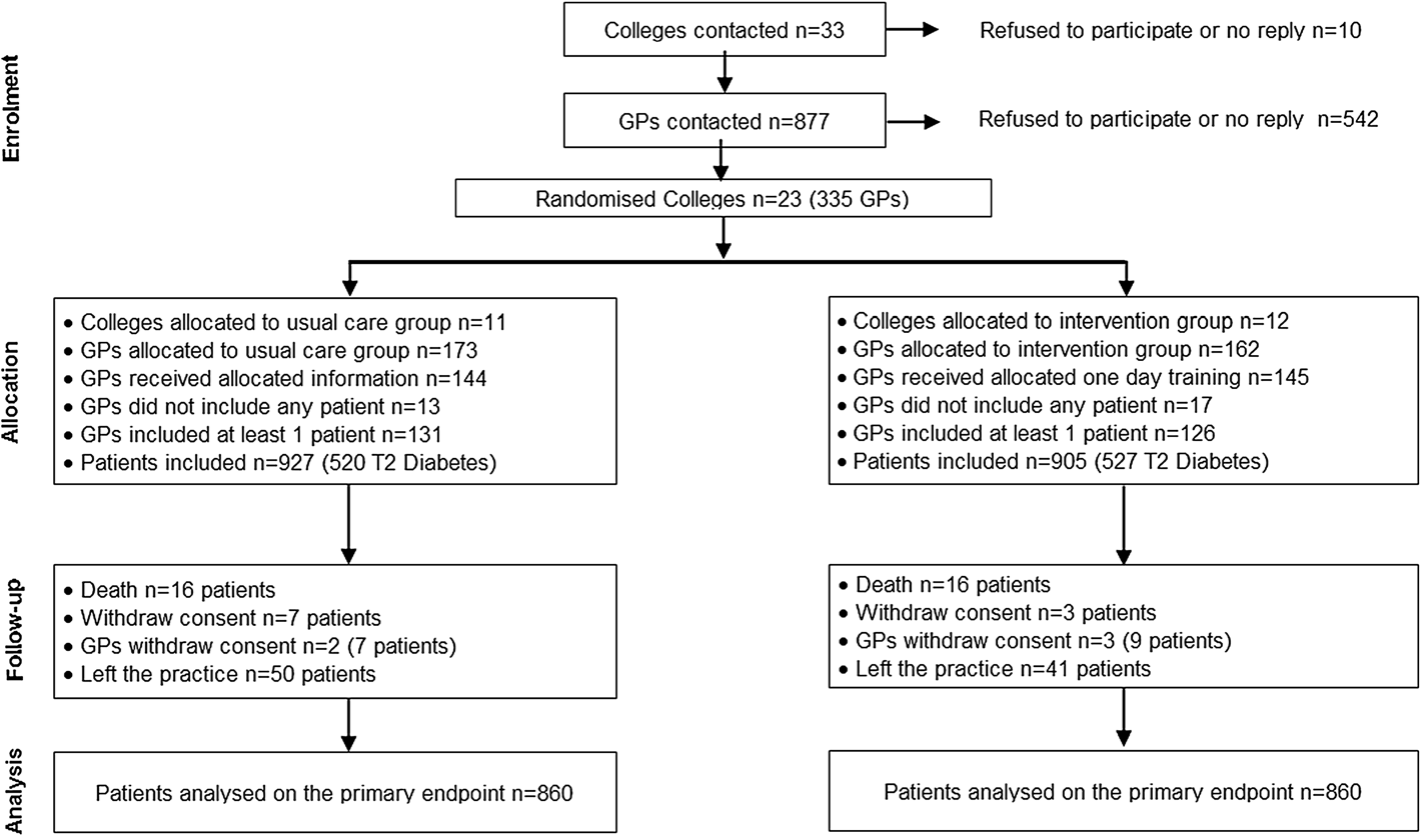
Flow of practices and patients through study.

Eleven colleges (173 GPs) were randomized to the usual care group and 12 (162 GPs) to the intervention group. Attendance at the one-day training in the intervention group or the 90 minute-meeting in the usual care group was mandatory for GPs to include patients. One hundred and forty-five GPs (90%) in the intervention group attended the one-day training, of which 126 (87%) recruited at least one patient in the trial. One hundred and forty-four GPs (83%) in the usual care group attended the 90 minute-meeting, of which 131 (90%) included at least one patient (Figure 1). The characteristics of the active GPs were similar in both groups in terms of gender, age, type and duration of practice (data not shown). The mean number of patients recruited per GP was 7.1 (minimum = 1, maximum = 16). Between November 2006 and July 2007, 1,832 patients were included in the trial, 927 in the usual care group and 905 in the intervention group. On average, they were 62 (SD 7.8) years old, and the sex ratio of men to women was 2:1. All patients were in primary prevention, had been treated for hypertension for an average of 10.9 years (SD 8.1), and 71% had more than two other cardiovascular risk factors associated with hypertension. The average body mass index was 30.5 kg/m^2^. The average diabetes duration of the 1,047 patients with type-2 diabetes was 7.5 years (SD 6.5).

At baseline (Table 1), patient characteristics were comparable in both groups, except for SBP and DBP, which were significantly higher in the intervention group by 7 mmHg and 3 mmHg, respectively (*P* < 0.001). In addition, significantly fewer diabetic patients had a prescription for aspirin in the usual care group compared with the intervention group: 26.9% versus 42.7% (*P* < 0.001). Finally, the percentage of patients achieving all of their therapeutic targets at baseline was significantly lower in the intervention group: 6.2% versus 10.2% (*P* = 0.005).

**Table 1:** Baseline characteristics of patients

**Characteristic**	**Intervention (n = 905)**	**Usual care (n = 927)**
Male, n (%)	575 (63.5)	589 (63.5)
Mean age, years (SD)	62.1 (7.9)	62.4 (7.7)
Height, cm (SD)	166.9 (9.3)	167.4 (9.1)
Weight, kg (SD)	85.8 (16.5)	85.6 (15.9)
Body Mass Index, kg/m^2^ (SD)	30.7 (5.2)	30.5 (5.0)
Men with waist ≥ 102 cm, n (%)	358 (62.8)	385 (66.3)
Women with waist ≥ 88 cm, n (%)	279 (85.6)	278 (85.3)
Mean systolic blood pressure, mmHg (SD)^a^	145.9 (15.3)	138.7 (13.5)
Mean diastolic blood pressure, mmHg (SD)^a^	83.7 (11.7)	80.6 (9.2)
Heart rate, beats/min (SD)	71.4 (10.9)	71.6 (10.4)
Total cholesterol, mmol/l (SD)	5.27 (1.15)	5.29 (1.14)
HDL, mmol/L (SD)	1.30 (0.35)	1.32 (0.37)
LDL, mmol/L (SD)	3.19 (1.02)	3.21 (1.04)
Triglyceride, mmol/L (SD)	1.84 (1.19)	1.78 (1.00)
Creatininemia μmol/L (SD)	86.2 (29.9)	85.4 (25.5)
MDRD-estimated glomerular filtration rate, ml/min (SD)	79.6 (19.6)	80.8 (20.2)
Fasting glycemia, mmol/l (SD)	6.88 (1.94)	6.77 (1.94)
Left ventricular hypertrophy, n (%)	150 (16.6)	184 (19.9)
Family history of early cardiovascular event, n (%)	225 (24.9)	253 (27.3)
Albuminuria ≥ 20 mg/L, n (%)	186 (22.3)	154 (18.1)
Mean years since diagnosis of hypertension (SD)	10.5 (7.8)	11.2 (8.3)
**Smoker status**		
Current n (%)	193 (21.3)	217 (23.4)
Past smoker < three years n (%)	72 (8.0)	94 (10.2)
Non-smoker n (%)	640 (70.7)	615 (66.4)
Number of antihypertensive drugs, n (SD)	2.16 (1.04)	2.18 (1.04)
Type-2 diabetes, n (%)	527 (58.2)	521 (56.2)
Mean years since diagnosis of type-2 diabetes, (SD)	6.9 (6.1)	7.8 (6.9)
HbA1c, % (SD)	7.0 (1.1)	7.0 (1.2)
Cardiovascular risk factors, n (%)		
Men > 50 or women > 60 years old	779 (86.1)	802 (86.6)
Current smoker or past smoker < three years	265 (29.3)	311 (33.6)
LDL ≥ 4.14 mmol/L or treatment	692 (76.5)	686 (74.0)
HDL ≤ 1.04 mmol/L	189 (20.9)	190 (20.6)
Number of cardiovascular risk factors, n (%)		
≤ 2	259 (28.6)	264 (28.5)
3	319 (35.2)	315 (34.0)
4	222 (24.5)	247 (26.6)
≥ 5	105 (11.6)	101 (10.8)
Ten-year Framingham-Anderson risk score, (%)	17.5	17.0
Achieved all therapeutic targets (3 or 5), n/N (%)^a^	56/900 (6.2)	94/923 (10.2)
Achieved blood pressure target, n (%)^a^	207 (23.0)	392 (42.6)
Achieved LDL target, n (%)	370 (41.1)	395 (43.4)
No smoking, n (%)	712 (78.7)	709 (76.6)
**Patients with diabetes**		
Achieved HbA1c target n/N (%)	312/527 (60.1)	316/521 (61.7)
Low-dose aspirin (yes) n/N (%)^a^	225/527 (42.7)	140/521 (26.9)

^a^statistically significantly different between groups (*P* < 0.001).

*MDRD*, modified diet in renal disease; *SD*, standard deviation.

### Primary endpoint

Due to missing values at every time point, the primary endpoint could not be modeled for six patients; therefore, the analysis for the primary endpoint was based on 1,826 patients. Over two years of follow-up, the proportion of patients achieving all of their therapeutic targets increased in both groups, but the increase was significantly higher in the intervention group: OR 1.89, 95% CI 1.09 to 3.27, *P* = 0.024 (Table 2). Similar trends were observed in patients with and without type-2 diabetes, but did not achieve statistical significance.

**Table 2:** Primary outcome: changes within groups and differences between groups at month 24 (M24) in the proportion of patients achieving all their therapeutic targets

**Endpoint**	**Group**	**M0 *n*/N (%)**	**M24 *n*/N (%)**	**OR (95% CI) for within group comparison**	***P***	**OR (95% CI) for between group comparison**	***P*-value**
All patients (3 or 5 targets)	Intervention	56/900 (6.2)	110/860 (12.8)	3.23 (2.12 to 4.94)	< 0.001	1.89 (1.09 to 3.27)	0.024
	Usual care	94/923 (10.2)	118/860 (13.7)	1.71 (1.19 to 2.47)	0.004		
Hypertension + T2D (5 targets)	Intervention	7/527 (1.3)	24/526 (4.6)	3.90 (1.60 to 9.52)	0.003	2.36 (0.68 to 8.18)	0.175
	Usual care	9/520 (1.7)	14/513 (2.7)	1.65 (0.69 to 3.98)	0.262		
Hypertension (3 targets)	Intervention	49/373 (13.1)	86/334 (25.8)	3.12 (1.94 to 5.03)	< 0.001	1.63 (0.99 to 3.01)	0.120
	Usual care	85/403 (21.1)	104/347 (29.0)	1.91 (1.27 to 2.88)	0.002		

*CI*, confidence interval; *M*0, month 0; *M24*, month 24; *T2D*, type-2 diabetes; *OR*, odds-ratio.

### Secondary endpoints

#### Individual therapeutic targets

The proportion of patients achieving their BP targets did not change significantly in the usual care group. However, significantly more patients in the intervention group achieved their BP targets at two years. The difference between the two groups was significant: OR 2.03, 95% CI 1.44 to 2.88, *P* < 0.001 (Table 3). The proportion of patients achieving their targets for LDL and quitting smoking increased in both groups, with no significant difference between the groups. There was no change in the proportion of patients with HbA1c ≤ 7% in either group in the type-2 diabetes sub-population.

**Table 3:** Within group and between group differences in the percentages of patients achieving their individual therapeutic targets

**Target**	**Group**	**M0 *n*/N (%)**	**M24 *n*/N (%)**	**OR (95% CI) within groups**	***P*-value**	**OR (95% CI) between groups**	***P*-value**
Blood pressure^a^	Intervention	207/900 (23.0)	303/823 (36.8)	2.55 (1.96 to 3.30)	< 0.001	2.03 (1.44 to 2.88)	< 0.001
	Usual care	392/923 (42.6)	382/825 (46.3)	1.25 (0.99 to 1.58)	0.060		
LDL cholesterol^b^	Intervention	370/884 (41.9)	458/793 (57.8)	2.65 (2.05 to 3.41)	< 0.001	1.25 (0.88 to 1.78)	0.205
	Usual care	395/910 (43.4)	435/778 (55.9)	2.11 (1.65 to 2.71)	< 0.001		
No smoking	Intervention	712/905 (78.7)	664/804 (82.6)	3.75 (1.92 to 7.30)	< 0.001	0.81 (0.41 to 1.60)	0.550
	Usual care	709/926 (76.6)	659/808 (81.6)	2.98 (1.81 to 4.93)	< 0.001		
HbA1c^c^	Intervention	312/519 (60.1)	275/472 (58.3)	0.81 (0.58 to 1.16)	0.257	0.77 (0.47 to 1.27)	0.310
	Usual care	316/512 (61.7)	279/452 (61.7)	1.06 (0.74 to 1.53)	0.747		
Low-dose aspirin	Intervention	225/527 (42.7)	318/527 (60.3)	5.55 (3.61 to 8.54)	< 0.001	2.28 (1.27 to -4.09)	0.006
	Usual care	140/521 (26.9)	179/521 (34.4)	2.43 (1.57 to 3.77)	< 0.001		

*CI*, confidence interval; *M0*, month 0; *M24*, month 24; *OR*, odds-ratio.

^a^ ≤ 140/90 mmHg or ≤ 130/80 mmHg for type-2 diabetic patients.

^b^ ≤ 3.36 mmol/l or 2.59 mmol/l for type-2 diabetic patients.

^c^ ≤ 7%.

The proportion of patients with type-2 diabetes who received a prescription of low-dose aspirin increased in both groups, the increase in the intervention group being significantly higher than in the usual care group: OR 2.28, 95% CI 1.27 to 4.09, *P* = 0.006.

#### Other endpoints

The changes in the main risk factor parameters are summarized in Table 4. SBP was reduced by 1.2 mmHg in the usual care group and by 6.0 mmHg in the intervention group. The 4.8 mmHg absolute difference between the two groups was statistically significant, in favor of the intervention group (*P* < 0.001). Similarly, for DBP, the absolute difference between the groups at the end of the trial was 1.9 mmHg, statistically significant in favor of the intervention group (*P* < 0.002).

**Table 4:** Changes in the main clinical and biological parameters between month 0 and month 24

**Variable**	**Group**	**Month 0 Mean (SD)**	**Month 24 Mean (SD)**	**Estimated change over 24 months (SE)**	**Estimated difference ( *P *-value)**
Systolic blood pressure (mmHg)	Intervention (*n* = 905)	145.9 (15.3)	139.6 (14.6)	-6.00 (0.46)	-4.76 (< 0.001)
	Usual care (*n* = 927)	138.7 (13.5)	137.2 (12.6)	-1.24 (0.48)	
Diastolic blood pressure (mmHg)	Intervention (*n* = 905)	83.7 (11.7)	80.2 (10.4)	-3.32 (0.38)	-1.88 (< 0.001)
	Usual care (*n* = 927)	80.6 (9.2)	79.2 (8.0)	-1.44 (0.38)	
LDL cholesterol (mmol/L)	Intervention (*n* = 905)	3.18 (1.03)	2.82 (0.85)	-0.31 (0.03)	0.05 (0.075)
	Usual care (*n* = 927)	3.21 (1.03)	2.92 (0.91)	-0.26 (0.03)	
MDRD-estimated GFR (ml/min)	Intervention (*n* = 905)	79.6 (19.6)	80.6 (20.1)	1.52 (0.48)	2.72 (< 0.001)
	Usual care (*n* = 927)	80.8 (20.2)	78.9 (20.5)	-1.20 (0.48)	
Framingham-Anderson Score (%)	Intervention (*n* = 905)	17.47	15.24	-2.23	0.06 (0.001)
	Usual care (*n* = 927)	17.00	15.81	-1.19	
Weight (kg)	Intervention (*n* = 905)	85.8 (16.5)	85.5 (16.8)	-0.28 (0.12)	-0.12 (0.470)
	Usual care (*n* = 927)	85.6 (15.9)	85.4 (16.1)	-0.16 (0.12)	
Waist circumference (cm)	Intervention (*n* = 905)	105.1 (12.8)	104.9 (13.3)	-0.08 (0.16)	-0.24 (0.269)
	Usual care (*n* = 927)	105.1 (13.3)	105.3 (13.8)	0.16 (0.16)	
HbA1c (%)	Intervention (*n* = 527)	7.01 (1.12)	7.03 (1.10)	0.03 (0.04)	0.01 (0.341)
	Usual care (*n* = 521)	7.01 (1.23)	7.03 (1.13)	0.02 (0.04)	

*LDL*, low-density lipoprotein; *MDRD*-*estimated GFR*, MDRD-estimated glomerular filtration rate; *SD*, standard deviation; *SE*, standard error. *P*-values refer to the comparison of the changes between the intervention and usual care groups in a three-level hierarchical generalized linear model (see Statistics section).

At baseline, the Framingham-Anderson scores were comparable in the two groups. In absolute values, this score decreased by 1.2% in the usual care group and by 2.2% in the intervention group. The difference between the two groups was statistically significant in favor of the intervention group (*P* < 0.001).

#### Sensitivity analysis of blood pressure

Restricting the analysis to centers that measured blood pressure with an automatic device (900 patients in the intervention group and 248 in the usual care group) gave similar results, with a significant difference in favor of the intervention group for the change in systolic (3.5 mmHg, *P* = 0.001) and diastolic blood pressure (1.3 mmHg, *P* = 0.045) over time.

#### Analysis of changes in blood pressure adjusted on baseline values

We have also analyzed the blood pressure data with an adjustment on baseline values to assess the extent to which the differential changes observed during follow-up were dependent of the between-groups difference at baseline. The results still demonstrate a significant effect of the intervention. For SBP, time effect was significant (*P* = 0.018) and the intervention effect (*P* < 0.0001) and the interaction between group and time were also significant (*P* < 0.0001). The adjusted difference over two years of follow-up was 4.8 mmHg. For DBP, time effect was significant (*P* = 0.0003) and the intervention effect (*P* < 0.0001) and the interaction between group and time were also significant (*P* < 0.0001). The adjusted difference over two years of follow-up was 1.9 mmHg.

#### Antihypertensive drugs

At baseline, the average number of antihypertensive drugs per patient was similar in the two groups, 2.16 (SD 1.04) in the intervention group and 2.18 (SD 1.04) in the usual care group. After two years, this number increased in both groups but increased significantly more in the intervention group: 2.41 (SD 1.05) versus 2.29 (SD 1.06) in the usual care group, (*P* = 0.020). In addition, significantly more patients in the intervention group received at least one additional antihypertensive drug over the two-year study period than in the usual care group (*P* = 0.009) (Table 5).

**Table 5:** Number of patients with a least one change in the number of antihypertensive drugs

	**Intervention, *n*** **= 812, n (%)**	**Usual care, *n*** **= 804, n (%)**	***P*-value**
Reduction	72 (8.9)	91 (11.3)	
No change	510 (62.8)	541 (67.0)	0.009
Increase	230 (28.3)	175 (21.7)	

#### Cardiovascular events

During the study, 89 cardiovascular events were reported, and 61 were validated using the patient records by a committee blind to randomization. The incidence of the first cardiovascular event was 3.0% in the intervention and 3.7% in the usual care groups, (*P* = 0.513).

#### Quality of life

Changes in scores for physical and mental quality of life were very small and not significantly different between the two groups (Table 6).

**Table 6:** Quality of life measured using the Short-Form-8 quality of life questionnaire (SF-8) tool at month 0 and month 24

**Quality of life**	**Group**	**M0**	**M24**	***P *M0-M24**	***P*-value**
SF-8 PCSS (SD)	Intervention (*n* = 872)	47.1 (8.3)	46.3 (8.7)	0.01	0.823
	Usual care (*n* = 868)	46.8 (8.6)	46.3 (8.9)	0.03	
SF-8 MCSS (SD)	Intervention (*n* = 872)	47.7 (9.4)	48.6 (9.2)	0.006	0.374
	Usual care (*n* = 868)	47.4 (9.8)	47.8 (9.3)	0.13	

*M*, month 0; *M24*, Month 24; *MCSS*, mental component summary score; *PCSS*, physical component summary score.

## Discussion

ESCAPE is the first interventional cluster randomized trial targeted at GPs only, showing that an easy-to-perform multifaceted intervention, significantly increased the proportion of high-risk hypertensive patients in primary prevention who achieved their recommended therapeutic targets without adversely affecting their quality of life. This high-risk hypertensive French population was chosen because their hypertension and other cardiovascular risks are usually very poorly controlled [5,9,10]. The intervention tested in the ESCAPE trial showed its efficacy for the primary endpoint in the overall population, but not in the two sub-populations with or without type-2 diabetes taken apart - probably due to a lack of power, the results were consistent. These results were obtained on an ambitious primary endpoint and in spite of a significant improvement in the usual care group, the well-known Hawthorne effect [23]. The results from previous clinical trials in which the intervention was not targeted exclusively at GPs failed to show such an impact from the tested intervention [15-18].

The main impact of the intervention was principally due to the proportion of patients achieving their blood pressure targets and the increased prescription of low-dose aspirin for patients with type-2 diabetes. The other components of the primary outcome also improved significantly within each group (except for HbA1c), but their change over time was comparable between the two groups. In addition, the quality of life results suggest that there was no 'price to pay’ for a more intensive management to reduce cardiovascular risk in terms of quality of life. Although the study was not powered to produce significant results in the two sub-populations, it showed consistent effects in both subgroups.

The absolute difference in the reduction of SBP of about 5 mmHg in the intervention group was clinically relevant because this difference could be expected to reduce stroke mortality by 20%, and mortality related to cardiac ischemic events or long-term overall cardiovascular mortality by 15% [24].

In the ESCAPE trial, the percentage of patients achieving all their therapeutic targets at baseline was very low overall (8.2%), and even lower for patients with type-2 diabetes (1.5%). This raises questions, not only about the quality of care provided by the GPs regarding the practice guidelines, but also about the accessibility and relevance of the therapeutic targets recommended for primary care settings.

In this trial, the choice of an intervention focused on GPs only reflected the organization of the French health care system. Unlike many other European countries, there are no other healthcare professionals such as nurses, assistants, nutritionists or psychologists in French GPs’ offices.

In 2006, the French guidelines [2] recommended the prescription of low-dose aspirin in primary prevention for patients with type-2 diabetes and at least two other cardiovascular risk factors (high-risk patients). In the following years, the results from several trials failed to support this recommendation [25,26]. Despite this, the absolute increase for the prescription of low-dose aspirin was 7.5% in the usual care group and 17.5% in the intervention group.

The selection of similar groups of patients is never guaranteed in a cluster trial. However, in ESCAPE, 23 clusters were randomized, and the patients were included by as much as 257 investigators, which favored the constitution of comparable groups. In fact, patients were very similar regarding age, sex, weight, waist circumference, creatinine clearance, blood lipids, and smoking.

It is likely that both the higher blood pressure and the higher prescription of low-dose aspirin seen at baseline in the intervention group were due to the intervention itself. During the training seminar, prior to the inclusion of patients, the GPs were given an electronic measurement device and were asked to report the exact values on the case report forms, which almost certainly improved the accuracy of their BP measurements by reducing substantially end-digit preference [27,28]. The BP was shown to be underestimated in the usual care group because of a much higher end-digit preference [27]. GPs tend to round down BP results to the lower 0 mmHg or 5 mmHg values systematically because it is considered better for the physician, as well as for the patient, to have a lower value under treatment. In the absence of any recommendation to report blood pressure values as read in the usual care group, there is no reason why the measurement bias observed in this group would change during follow-up, and the comparison of the slopes of the changes in blood pressure over time remains valid. The effect of the intervention being tested as the interaction between time and the randomization group, the differences observed between the two groups at baseline could have only a small influence on the results, and this was confirmed by the results of the analysis adjusted on baseline values.

Moreover, restricting the analysis to centers that used the same method of blood pressure measurement gave results that still demonstrated a steeper decline in blood pressure in the intervention than in the usual care group. The 7/3 mmHg BP differences seen in ESCAPE are similar to that shown in the CRAB study [28] that compared the two blood pressure measurement methods in use in ESCAPE. The automatic blood-pressure device was part of the intervention, which explains why it was not provided to the usual care group. The only way to avoid the influence of the blood-pressure measurement device on the baseline blood pressure measurements in the intervention group would have been the recruitment of all the study patients before cluster randomization, a recommended method in cluster trials. This was not possible, however, for practical and financial reasons. In fact, patient recruitment took place over nine months. Moreover, if the baseline measurements had been performed before randomization, blood pressure would have risen spuriously at the first post-randomization visit in the intervention group, and the effect of the intervention would have been confounded by the Hawthorne effect.

During the training seminar, intervention group GPs were also reminded to prescribe low-dose aspirin to patients with type-2 diabetes. Due to the delay between this seminar and the inclusion of the first patients, some GPs have probably prescribed low-dose aspirin to patients with type-2 diabetes before including them into the ESCAPE trial.

Finally, the significant increase in MDRD renal blood flow in the intervention compared with the usual care group can be considered as a proof of a genuine improvement in cardiovascular risk management. In fact, because this parameter was not calculated using blood pressure values, results reflect real and not spurious changes in cardiovascular management.

Since the ESCAPE trial began, several randomized cluster trials with hypertensive patients or patients at high cardiovascular risk have been published. What makes ESCAPE original is that the intervention was targeted exclusively at GPs, and that it involved only hypertensive patients at high cardiovascular risk in primary prevention. EUROACTION [29] assessed an intervention delivered by nurses to patients at high cardiovascular risk and their families in primary care. The results showed a significant increase in the proportion of patients with a BP below 140/90 mmHg in the intervention group. However in EUROACTION, the intervention, the effectors (nurses), the people exposed to the intervention (patients and families) and the primary endpoint were different from those in ESCAPE since the patients in ESCAPE had a different profile and their family members were not directly exposed to the intervention. In the SPHERE trial [30], the intervention was complex, multifactorial, with multi-effectors (nurses and GP university lecturers) and multi-targets (nurses and GPs in practice). In addition, the patients included into the trial were all treated for secondary prevention. A significant reduction in the proportion of patients hospitalized for cardiovascular events was reported, without a significant increase in the proportion of patients achieving their blood pressure and total cholesterol targets. However, unlike the patients in ESCAPE, those included in the SPHERE trial were particularly well-controlled at inclusion, which led to a ceiling effect. In the COM99 trial, the intervention, which was targeted at hospital physicians and GPs, aimed to improve patients’ treatment adherence, their blood pressure control and to reduce the risk of cardiovascular events [31]. The physicians applied the intervention (motivational interviewing techniques) to patients with uncontrolled hypertension and a high cardiovascular risk (ten-year risk of 30%). The results from this trial showed that adherence and blood pressure control both improved but it did not appear to improve long-term cardiovascular events. This trial is not comparable with ESCAPE in terms of the population of physicians involved and the nature of the intervention and patients’ profile.

## Conclusions

An easy-to-perform, multifaceted intervention targeting only GPs increased significantly the proportion of high-risk hypertensive patients in primary prevention achieving their recommended therapeutic targets.

## Abbreviations

BP: Blood pressure; CI: Confidence interval; CNGE: French national college of teachers in general practice; DBP: Diastolic blood pressure; GFR: Glomerular filtration rate; GPs: General practitioners; HbA1c: Glycated hemoglobin; HDL: High-density lipoprotein cholesterol; LDL: Low-density lipoprotein cholesterol; MCSS: Mental component summary score; MDRD: Modified diet in renal disease; OR: Odds ratio; PCSS: Physical component summary score; SBP: Systolic blood pressure; SD: Standard deviation; SF-8: Short-Form-8 quality of life questionnaire.

## Competing interests

All authors have completed the Unified Competing Interest form at http://www.icmje.org/coi_disclosure.pdf (available on request from the corresponding author) and declare that (1) DP and DH had support from the French National College of Teachers in General Practice for the submitted work; (2) the authors have had no relationship with companies that might have an interest in the submitted work in the previous three years; (3) their spouses, partners, or children have no financial relationships that may be relevant to the submitted work; and (4) the authors have no non-financial interests that may be relevant to the submitted work.

## Authors’ contribution

DP conceived the study, wrote the protocol, supervised the study conduct, contributed to the data collection, and had a major role in writing the article. ML contributed substantially to the study design, was responsible for the study coordination and data management, and had a major role in writing the article. DH contributed substantially to the design of the study and to the data collection. FB designed and carried out the statistical analysis. JPL, VR, EB and XG contributed in interpreting the results and preparing the manuscript. All authors critically revised the article for important intellectual content and gave final approval for the version submitted. All authors read and approved the final manuscript.
